# Unknown unknowns: essential genes in quest for function

**DOI:** 10.1111/1751-7915.12384

**Published:** 2016-07-20

**Authors:** Antoine Danchin, Gang Fang

**Affiliations:** ^1^ Institute of Cardiometabolism and NutritionCHU Pitié‐Salpêtrière47 boulevard de l'Hôpital75013ParisFrance; ^2^ Department of BiologyNew York University Shanghai Campus1555 Century Avenue, Pudong New AreaShanghai200122China

## Abstract

The experimental design of a minimal synthetic genome revealed the presence of a large number of genes without ascribed function, in part because the abstract laws of life must be implemented within *ad hoc* material contraptions. Creating a function needs recruitment of some pre‐existing structure and this reveals kludges in their set‐up and history. Here, we show that looking for functions as an engineer would help in discovery of a significant number of those, proposed together with conceptual handles allowing investigators to pursue this endeavour in other contexts.

Natural selection, as a physical process, has implanted within living cells a way to organize an inherent lack of predictability. It allows them to cope with this unavoidable stumbling block. To be sure, among the certainties that might illuminate year 2020 is the fact that yet another unexpected discovery will change our views about life. Yet, we can get some plausible predictions as a more or less smooth follow‐up of what is already under investigation. Here, using the point of view of the engineer, we explore the constraints that operate on autonomous cell life, starting with the construction of the synthetic cell designed by the J. Craig Venter Institute (JCVI) recently (Hutchison *et al*., 2016). The basis of our predictions rests on functional analysis, an approach that places functions first, before considering the structures that drive them in action (Acevedo‐Rocha *et al*., 2013). Engineering knowledge allows us to identify functions that need to be present in any such construct. We end this presentation with exploration of some of those in a variety of bacterial clades.

To create synthetic autonomous bacteria, the JCVI designed and produced a *Mycoplasma mycoides*, streamlined strain JCVI‐syn3.0 (Syn3.0), comprising 473 genes within a genome 531 kb long (Hutchison *et al*., 2016). A remarkable claim from this work was that some 149 genes with unknown function (84 labelled “generic” and 65 “unknowns”) were essential to allow the cells to produce viable colonies on plates supplemented with highly nutritious mixtures (Collaboration, 2015). The surprising find was the sheer number of unknowns, one‐third of the genome. This figure is far higher than what could be expected from previous studies. Will we be able to identify these unknown functions by year 2020, and why is this so important?

## Streamlining a genome to its paleome component

Anticipating discoveries asks for a consistent understanding of what a living cell entails. We use here the view that cells are akin to computers, not those we know, but computers that would create a progeny of computers (Danchin, 2009c). Briefly, a computer associates a concrete machine and a physically separate programme. A genome program, as a computer program, can be split into an operating system and a variety of applications. In line with this view, the SynBio efforts are split between designing the minimal program for a universal machine, and specific applications, such as metabolic engineering design. This split corresponds to two functional units: a first unit (the paleome) is common to all members of a given species; a second one (the cenome) specifies occupation of a niche by the member strains of the species (Acevedo‐Rocha *et al*., 2013). The JCVI effort attempted to design a minimal instance of the former, building up a streamlined paleome of *M. mycoides*, with a first pruning process that got rid of more than half of the original genome sequence (Hutchison *et al*., 2016) that which coded for the cenome of the chosen strain and perhaps some dispensable functions from the paleome (e.g. YqeH, a K^+^‐dependent NTPase associated to the ribosome and involved in subunit 30S maturation, MMSYN1_0488; Rafay *et al*., 2012).

Most synthetic biology efforts such as that of the JCVI do not deal with the machine itself (which is taken for granted), but only with the conceptual and physical design of the program. It is unlikely that this particular situation will be much changed by year 2020. However, the practical building up of programs will have evolved, in particular with the undemanding genome editing techniques derived from the CRISPR‐Cas9 craze (Bates, 2016). Furthermore, there will be progress in the construction of genomes with nucleotides differing from the standard deoxyribonucleotides: *Escherichia chlori* replaced thymine by 5‐chlorouracil in *Escherichia coli* (Marliere *et al*., 2011), while four non‐canonical nucleotides were shown to polymerize *in vivo* (Eremeeva *et al*., 2016). Here, we identified many of the unknown functions of the Syn3.0 construct, and give directions for future functional developments of minimal cell machines (named “chassis” in the private jargon; de Lorenzo and Danchin, 2008).

A key feature of the computer model is that it is an abstract design. In terms of the matter involved when building up a concrete instance of the machine, it is not important that its core microprocessor is made of silicium or of gallium arsenide, nor that it is a PC or a Mac. Hence, while the concept of the machine is universal, its concrete implementation follows the idiosyncrasies of history or of the matter used in its construction. This somewhat unfortunate feature – this means that sometimes matter or history, not function, input their mark, producing fairly anecdotal features – is well illustrated in the Syn3.0 construct. To set the stage while emphasizing the contribution of history, we identified for one of its unknown unknowns a quick and dirty solution that remained essential in a particular chassis lineage: a target‐specific endoprotease, RppA/YsxB, cleaves off the nine N‐terminal residues of ribosomal protein L27 (involved in ribosome assembly and peptidyl transferase catalysis) to make it functional. This kludge has been observed in Firmicutes (and in the derived Tenericutes, to which *M. mycoides* belongs). This protease (MMSYN1_0500) is essential in *Staphylococcus aureus* (Wall *et al*., 2015), and persistent in *Bacillus subtilis* (Fang *et al*., 2005). Remarkably, it is expected that this gene/protein, absent from *E. coli* where L27 is not truncated, will be absent from non‐Firmicutes. This finding alone establishes that there is no universal biological chassis but a variety of concrete implementations with diverse prospects in terms of allowed actions within the large spectrum of possible environments (see below).

Another idiosyncrasy of Mycoplasmas, previously well documented, is that they use codon UGA to code for tryptophan, a feature not shared by the majority of bacterial clades (Ohama *et al*., 2008), but helpful when using standard chassis to construct a synthetic genome from bits and pieces. This feature will impact genes involved in the translation machinery (as indeed observed in the Mycoplasma general set‐up; Grosjean *et al*., 2014). Moreover, and this is seldom highlighted, these organisms, as some Lactobacilli, do not use iron (Weinberg, 1997), a fact that has enormous consequences in terms of metabolism, sensitivity to reactive oxygen species and proton management. Finally, a further functional feature is again illustrated with Syn3.0. The functions may be universal but the actual genes’ descents may differ. As a case in point, degradation of very short oligonucleotides (nanoRNAs) is an essential cleaning process that copes with the leftovers created by ribonucleases. In gamma‐Proteobacteria (and mammals as well), this function is fulfilled by protein Orn and proteins of a common descent (Bruni *et al*., 2013). In Firmicutes, a functional (but not structural) counterpart is NrnA, also found in Mycoplasmas (Postic *et al*., 2012), but not identified in the Syn3.0 article. The “generic” gene/protein MMSYN1_0139 is the expected counterpart. All these features imply that the concrete set‐up of the Syn3.0 cell will differ from other designs based on chassis stemming from different bacterial clades.

A complete summary of our predictions is presented in Table S1. When identified, the protein names correspond to the ongoing annotation of the *Bacillus subtilis* genome at https://www.genoscope.cns.fr/agc/microscope/mage/viewer.php?O_id=7.

## Functional identification of Syn3.0 generic unknowns and unknown unknowns

We further split the paleome functions into a constructor, a replicator and a variety of functions, some of which presumably unknown but required for perennization of life. The functions of the constructor, comprising the machineries of translation and transcription, are fairly well established, with the ribosome as it core structure. Here, we propose identification of a significant number of relevant functions belonging to the class deemed unknown (Hutchison *et al*., 2016).

### RNA metabolism

Ribosome assembly requires many of these “difficult” functions. Processing, then folding long RNA molecules into a correct structure is not straightforward. In particular, as engineers know, correct distances between objects or catalytic sites must be measured. Molecular rulers are involved in the process (Zhang and Ferré d'Amaré, 2016). The RulR/YlxR‐RulQ/YlxQ couple (with conserved gene synteny in Firmicutes), with RulQ (MMSYN1_0298) a ruler binding to the widespread RNA structural motif RNA kink turns (K‐turns (Baird *et al*., 2012)), bends the helical trajectory of the RNA by 120°, associated to RulR/YlxR, MMSYN1_0299.

RNA turnover also involves a variety of functions both because messenger RNA is a labile molecule, thus allowing fine tuning of gene expression, and because even stable RNAs (ribosomal RNA and tRNA) have a finite lifetime, strongly dependent on proper folding (Phizicky and Hopper, 2010). CshB/DeaD (MMSYN1_0410) is a DEAD ATP‐dependent helicase, essential for DNA editing (it has a fairly conserved synteny with Nfo, involved in DNA metabolism, see below). It may be promiscuous in Syn3.0, allowing RNA degradation and possibly rRNA 23S maturation too.

Now, to degrade RNA in the 5′‐>3′ direction, the triphosphate end of newly synthesized mRNA must be cleaved off. The corresponding function is ubiquitous but structurally variable. In *B. subtilis*, this function is carried out by a protein of the family of nucleoside triphosphate pyrophosphatases (Piton *et al*., 2013) differing structurally from the *E. coli* enzyme RppH (Foley *et al*., 2015). Protein MMSYN1_0127, similar to YwfO from *B. subtilis*, belongs to this class and might perform this expected function (see however below). Less efficient, but adequate hydrolysis of the mRNA 5′ end triphosphate may also be found within the promiscuous phosphatases with unassigned activity present in the unknown genes. Ribonucleases are further essential components of the cell machinery. Firmicutes‐specific RNase J1 (RnjA, MMSYN1_0600 (Hutchison *et al*., 2016)) is present in Syn3.0, unexpectedly together with RnjB, found in a very limited number of bacterial clades (MMSYN1_0257). The detailed structure of the Firmicutes RNA degradosome components remains fairly elusive (Danchin, 2009a). It may comprise the previously discussed nanoRNase A, and also MMSYN1_0005, PgpH, a pApA phosphodiesterase, as well as exonuclease YhaM, MMSYN1_0437, the function of which is indeed ubiquitous in the Firmicutes/Tenericutes clades – but, due to its promiscuity, not yet correctly identified. Finally, the ultimate degradation products of RNA are often cyclic phosphodiesters which need to be opened up. We propose that MMSYN1_0431 is PdeB/YmdB, playing this important role. Subsequently, nucleotides may be recycled, or, in the case of modified nucleotides, degraded into modified bases that will be exported from the cell when they cannot be further metabolized. Nucleotide salvage entails a variety of functions, mainly phosphatases and kinases (see Table S1 and below).

### Translation

Most of the translation‐related functions are readily identified in Syn3.0, belonging to the minimal *Mycoplasma* sp. translation apparatus (Grosjean *et al*., 2014). A few remain to be characterized, such as protein EttA that adapts translation initiation and speed to energy availability (Boel *et al*., 2014). This protein is likely present as four paralogues in *B. subtilis* (YfmM, YkpA, YdiF and EttA/Uup). Protein EttM/YfmM (MMSYN1_0853) is a plausible counterpart in Syn3.0. Ribosomal RNAs also interact with more than 50 ribosomal proteins, which must bind in an orderly fashion. Furthermore, once folded, the ribosome must remain stable during the process of translation. Modification of RNA (essentially by methylation and sometimes via isomerization of uracil into pseudouracil) is involved in these processes.

We can already find a significant number of these activities in the Syn3.0 strain. Among those, beside highly conserved identified methyltransferases (Hutchison *et al*., 2016), one finds less prominent counterparts such as RsmI (MMSYN1_0504), methylating the ribose of a cytidine already methylated by RsmH, and we propose that MMSYN1_0838 is RlmB. Methylation of ribose is important to prevent formation of unwanted hydrogen bonds that would prevent a variety of non‐Watson–Crick base associations. This is important both for rRNA and tRNA proper folding. In half of the Mollicutes, there is another ribose 2′‐O‐methyltransferase, RlmBB/YsgA, MMSYN1_0448, which is present together with RlmB. Its exact function, in modification of rRNA or tRNA needs to be established experimentally. In the same way, many tRNA modifications could be identified (“equivalog,” “probable” and “putative” categories in the JCVI nomenclature), but several remained within the unknowns (“generic” category), such as, possibly A+T‐rich Firmicutes/Tenericutes‐specific TrmK (MMSYN1_0408), TrmNF (MMSYN1_0043), DusB (MMSYN1_0063), TsaE, which cooperates with other identified subunits (MMSYN1_0270), TruA (MMSYN1_0640) and the RNA‐binding protein YtpR, which might act as a methylase for nucleotide G6 of tRNA phenylalanine. Finally, in the category of maturation proteins, we identified protein RppA(YsxC) (MMSYN1_0500) as discussed previously – noting that the catalytic histidine and cysteine residues are perfectly conserved (Wall *et al*., 2015).

The process of loading aminoacids on tRNAs may result in unwanted activation of wrong aminoacids. Enigmatic protein HinT, MMSYN1_0438, is a ubiquitous aminoacyl‐adenylate hydrolase. This protein may also have other metabolic cleaning functions, possibly removing accidental coupling of nucleotides to proteins. It is a ubiquitous protein that should be submitted to thorough biochemical exploration.

Academic textbooks generally teach us that ribosome‐translated proteins contain 20 amino acids. This carries over a misleading view, as proline does not have a primary amino group. Indeed, this molecule was long fairly correctly described as an *imino* acid – this nomenclature has unfortunately become obsolete, concealing the special character of proline (NCBI, 2016) that makes its insertion within a polypeptide chain chemically challenging. To be sure, its inclusion in the chain depends upon a specific component of the translation machinery, translation factor EF‐P (Woolstenhulme *et al*., 2015). Syn3.0 does not escape this constraint (MMSYN1_0391 is EF‐P). Remarkably, to be functional, EF‐P further needs a post‐translational modification. In Syn3.0, the modified residue is not a lysine, as in *B. subtilis* or *E. coli*, but an arginine. This residue is altered by rhamnosylation in *Pseudomonas aeruginosa* and many other bacteria (Rajkovic *et al*., 2015). It is likely that one of the glycosyltransferases coded in the genome, such as MMSYN1_0697 will act in this way (there is a “putative” rhamnosyltransferase, MMSYN1_0114, but its counterparts are not similar to the Eag glycosyltransferase found in *Pseudomonas* sp.). Proline presence further requires specific protein folding features – and it is surprising that the ribosome‐bound trigger factor (Tig) could be deleted to yield the Syn3.0 construct. However, we remark that protein MMSYN1_0805 has some similarity with Tig so that it could play its molecular chaperone role, in particular as a proline isomerase.

Noting that proline polymerization is difficult, we confirmed that the Syn3.0 genome coded for a valine tRNA ligase (MMSYN1_0260) comprising a PPP tripeptide, as found in the vast majority of organisms. No other such tripeptide was found in the proteome, but there are several PP dipeptides that, because they are likely counterselected unless important for function of the cognate proteins, we used as probes to substantiate our predictions of unknown functions. Finally, proline polymerization likely results in premature translation termination, producing proline‐containing peptides that must be broken down. Protein MMSYN1_0305 could be protein PapA(YqhT), which fulfils this role (Zaprasis *et al*., 2013).

A similar requirement for degradation should prevail with aged proteins. Proteins age spontaneously, be it only because aspartate and asparagine isomerize into isoaspartate with a context‐dependent specific half‐life (Robinson and Robinson, 2004). In general, this will alter and possibly inactivate the protein function, asking for protein disposal. The cell copes with this situation in three ways: it exports aged proteins (the corresponding machinery, if it exists, is unknown; long‐term propagation of colonies of Syn3.0 would provide hints about its existence), discards proteins as aggregates that will remain within aged cells (Saarikangas and Barral, 2016), or cleaves aged proteins into peptides, then amino acids. This latter function asks for a variety of proteases and peptidases. When cytoplasmic, these proteases must be specific to avoid destroying functional proteins. Isoaspartate residues in the backbone polypeptide chains provide convenient tags for degradation. Cytoplasmic endopeptidase O (MMSYN1_0444), with its conserved HEXXH Zn^2+^‐binding motif, is a case in point. In the membrane, protein MMSYN1_0516 could possibly be the important endoprotease GlpG, with some but not all important residues conserved. While peptidases recognizing isoaspartate residues have been found in *E. coli*, no clear‐cut counterparts have been found in other bacterial clades. There is a variety of peptidases that may play the expected roles. Because this family of functions is likely to be highly promiscuous, several peptidases could be deleted from the parent of strain Syn3.0. However, several remain, such as PepA/YuiE (MMSYN1_0154), YtjP (MMSYN1_0493), with a conserved catalytic histidine and conserved PPG tripeptide and which could be a promiscuous peptidase/deacylase and MMSYN1_0133. This type of activity should therefore be on the priority list for the discovery of unknown functions in the next few years (Bohme *et al*., 2008).

Other proteases might be specifically involved in cherry‐picking aged counterparts of recently synthesized proteins in such a way as to produce a young progeny. We expect ATP‐dependent proteases to use ATP as a means to reset their function after they have targeted this age‐specific information (Binder and Danchin, 2011). In most organisms, this function is performed by functionally related proteins such as GroEL, DnaKJ, GrpE and Lon. DnaK, GrpE and Lon belong to the identified category in Syn3.0. GrpE is a nucleotide recycling factor, a family of proteins that are essential for resetting the activity of informational ATPases. Protein MMSYN1_0353 is a likely counterpart involved in division, but its associated ATPase is not known (the Syn3.0 proteome harbours several candidates). Besides these essential activities, a similar set of proteases is involved specifically in cell division. Membrane‐bound protein MMSYN1_0039 may display at least some of the ATP‐dependent proteolytic function of protein FtsH. It has a highly conserved ATP‐binding site, with a PPG‐conserved tripeptide.

### DNA metabolism

The chromosome structure and its involvement in cell division require several complexes, with subunits only partially identified among the known proteome. Overall stabilization of the chromosome is performed by HU proteins, a counterpart of which is MMSYN1_0350. Energy‐dependent factor SMC (MMSYN1_0415) colocalizes with its interacting partners, ScpA (MMSYN1_0327) and ScpB (MMSYN1_0328), and the specific localization of SMC depends on both Scp proteins, showing that all three components of the SMC complex are required for proper localization. Dimeric ScpB stabilized the binding of ScpA to the SMC head domains, substantiating our prediction that MMSYN1_0327 is ScpA. Besides proteins with identified function, cell division involves DnaD (MMSYN1_0352), WhiA/YhcL (MMSYN1_0817) and GpsB/DivIVA (MMSYN1_0353), a putative nucleotide recycling factor discussed previously.

Now, DNA replication needs repair and proofreading. Proofreading during repair is performed by a 5′‐3′ exonuclease, associated to repair polymerase I (PolA) in gamma‐Proteobacteria, but standing alone in many clades related to Firmicutes (Fukushima *et al*., 2007). This function is likely performed by protein ExnP/YpcP (MMSYN1_0097). It is linked to the need for removal of RNA or ribonucleotides in DNA (which would be performed by RNase H‐like protein YpeP, MMSYN1_0283), to excision of apurinic/apyrimidic sites (protein Nfo, MMSYN1_0109) and to repair of double‐strand breaks (RecN, MMSYN1_0388 and YfdD, MMSYN1_0511). A virus‐related dUTPase, DutX (MMSYN1_0447) will prevent U from getting in DNA. There is also a need for preventing as much as possible non‐standard deoxyribonucleotides to get into the chromosome. YwfO (MMSYN1_0127) would act as a promiscuous pyrophosphohydrolase on modified trinucleotides. Finally, it is expected that availability of deoxyribonucleoside triphosphates is a limiting factor, prompting salvage: we expect that MMSYN1_0382 is the required promiscuous deoxyribonucloside kinase (McElwain and Pollack, 1987).

### General metabolism

Lacking iron‐related metabolism, Mycoplasmas are restricted to fermentation processes. The cells need to compensate for lack of respiration‐linked vectorial transport of protons, possibly using ATP synthase in the reverse direction, hydrolysing ATP to export protons (Kobayashi *et al*., 1986; Mileykovskaya *et al*., 1987; Sakamoto *et al*., 2002). Protons will be used to build up an electrochemical gradient and to co‐transport metabolites. This has important consequences in the management of inorganic phosphate, a metabolism area where much remains to be understood. Hence, the cell must scavenge, store and salvage this mineral. Syn 3.0 hosts a large number of likely phosphatases with unknown specificity (MMSYN1_0066; MMSYN1_0710; MMSYN1_0907; HadM/YxeH alpha‐D‐glucose‐1‐phosphate hydrolase, promiscuous, MMSYN1_0077; RibZ/HadM, MMSYN1_0530). Interestingly, multiple poorly identified phosphatases exist also in *B. subtilis*, where four highly similar phosphatases have been identified, one of which hydrolyses 5‐amino‐6‐ribitylamino‐2,4(1H,3H)‐pyrimidinedione 5′‐phosphate and FMN (Sarge *et al*., 2015). This suggests that the exact function of these enzymes is not yet understood despite their importance, hidden by promiscuity. An important function could be detoxification of unwanted analogues of the glycolytic pathway metabolites, as recently demonstrated in eukaryotic cells (Collard *et al*., 2016).

The role of phosphate goes far beyond being the standard “quantum” of energy exchanged in metabolic processes. Inorganic phosphate regulates a large number of processes in all cells, in particular in relation with the synthesis and turnover of polyphosphate (Albi and Serrano, 2016). In parallel, pyrophosphatase (MMSYN1_0344) is the core enzyme that uses hydrolysis to drive forward metabolism as a whole (Danchin and Sekowska, 2014). It is not firmly established whether, as in all living organisms (Rao *et al*., 2009), Mycoplasmas produce inorganic polyphosphates (polyP). Syn3.0 contains a counterpart of the RelA/SpoT protein synthesizing (p)ppGpp (Hoelzle *et al*., 2010), but, as in the case of other Mycoplasmas, it does not code for a structural counterpart of PPX, the enzyme that hydrolyses polyP. Whether a protein with a different structure plays this function is not known, but this is quite possible as the functional counterpart of PPX in *S. cerevisiae*, for example, is essential but of a completely different descent (Sethuraman *et al*., 2001). PhoU is known to be involved in polyP synthesis or turnover but its explicit function is yet unknown (de Almeida *et al*., 2015). Interestingly, Syn3.0 possesses a highly conserved PhoU counterpart, MMSYN1_0428. Overall, these functional associations are in favour of the presence of polyP in the Mycoplasma minimal genome with ppGpp as a possible seed and PhoU as a component of the machinery.

Fermentation is essential for generating energy in Syn3.0, and the result of the pathway will be production of reduced equivalents. A significant fraction of the enzymes that may equilibrate the pools of reduced and oxidized NAD could be deleted during the construction of Syn3.0. However, MMSYN1_0302 is a likely NAD(P)H water‐forming oxidase, possibly promiscuous, that would help equilibrate the redox pool of metabolites (Shi *et al*., 2016).

### Chemical stresses

The glycolysis/fermentation pathways are tightly coupled to phosphate turnover. Phosphorylated analogues of the corresponding three‐carbon intermediates are both essential for some syntheses but toxic when in excess. As an example, erythrose‐4‐phosphate inhibits phosphoglucose isomerase (Pgi) in plants (Zhou and Cheng, 2008) as well as in some types of fermentative organisms (Richter *et al*., 2003). Two enzymes may thus be inhibited in Syn3.0, Pgi (MMSYN1_0445) and possibly ManA (mannose‐6‐phosphate isomerase, MMSYN1_0435). The phosphatases just discussed may participate in detoxification. However, this may result in a trade‐off: besides reducing sugars, known to impact proteins via age‐dependent glycation, several glycolysis intermediates become reactive compounds when dephosphorylated. Methylglyoxal is a case in point. This molecule reacts with a variety of protein or nucleic acid amino groups, requiring specific protection. The *E. coli* glyoxalase SufL (YraA in *B. subtilis*) is involved in adaptation to multiple stresses via protection against methylglyoxal toxicity via deglycation of proteins (Chandrangsu *et al*., 2014). MMSYN1_0400 is the likely counterpart in Syn3.0.

Finally, the absence of iron metabolism in Mycoplasmas protects them against the most crucial deleterious effects of dioxygen. However, a variety of metabolic reactions are likely to produce reactive oxygen species that will have several targets, in particular cysteine residues. This may explain why a peroxiredoxin counterpart may be essential: MMSYN1_0054 is a candidate to play the role of *B. subtilis* peroxiredoxin YgaF.

### Transport

The cytoplasmic membrane insertion machinery is well conserved in Syn3.0. Here we suggest to further include MMSYN1_0430 as the counterpart of YlxM, a Firmicute/Tenericute component of the signal recognition particle protein membrane‐targeting pathway, which might interact with 4.5S RNA (Williams *et al*., 2014).

Influx of essential metabolites and efflux of waste or toxic material are essential to the cell function. A major proportion of the unknowns listed in the description of Syn3.0 corresponds to membrane proteins similar to transport proteins, albeit without clearly assigned function. We could not, in any straightforward way, identify the specific function of a majority of those. Nor were we able to predict whether they control influx or efflux. However, we list here several instances where similarity with known transporters is significant enough to allow us to propose a function.

Magnesium is an essential ion, and a magnesium transporter has been readily identified in Syn3.0 (MMSYN1_0787). Other divalent metal ions must also enter the cell, and, in the absence of protection against reactive oxygen species, Lactobacilli (that share the lack of iron specific to Mycoplasmas) use divalent manganese as a mineral scavenger of superoxide (Culotta and Daly, 2013). We propose that membrane protein MMSYN1_0879 is the required manganese permease. This protein has many features in common with a previously recognized magnesium transporter, and manganese is an excellent mimic of magnesium. With no iron, Mycoplasmas do not need corresponding chelators or permeases. However, they use zinc, which binds to sites often quite similar to iron binding sites (hence making predictions from the sequence difficult). Zinc is present in the environment at a low concentration: the cells need both transporters and binding proteins allowing storage. Among the unknown proteins of Syn3.0, we found at least two components that might be involved in the process. Widely conserved TIM barrel protein CutC, MMSYN1_0433, is unlikely to bind copper because it lacks one of the essential cysteine residue. However, because it keeps many of the other important residues, it may be involved in binding another metal, possibly zinc. In the same way, protein MMSYN1_0620, similar to Zur in *B. subtilis*, could be involved in controlling zinc capture and/or processes associated to zinc imbalance, such as production of hydrogen peroxide, via regulation of gene expression. Besides magnesium, polyamines are essential in all processes asking for stabilization of nucleic acids, as well as in accuracy of translation. Their biosynthesis is not encoded in the genome but there are counterparts for a potent spermidine/putrescine transport system: PotA, MMSYN1_0197, ATP‐binding subunit, PotB, MMSYN1_0196 permease subunit and PoCXD, MMSYN1_0195, permease + binding subunit, a fusion protein with an internal domain, PotX, of unknown function.

Finally, there is a need for lipid transporters, in particular for equilibration of the inside and outside membrane in terms of the quality of their phospholipids that may be involved in the membrane curvature and in the formation of lipid rafts important for membrane fluidity (Bramkamp and Lopez, 2015). YwjA, MMSYN1_0371, is a lipid transporter/flippase ABC transporter combining an ATP‐binding site and a permease within a single polypeptide that might fit the bill.

## Perspective: beyond the Tenericutes clade

Besides formation of a membrane separating the inside from the outside, two nanomachines make the core of living cells. The ribosome translates RNA and ATP synthase, associated to chemiosmotic laws, manages energy. Based on the principle of dichotomy, a so‐called “tree of life” is built up using the descent of ribosomal RNA as its reference sequence. In general, the tree of ATP synthase subunits (at least some of them) is congruent to that of ribosomal RNA. Yet, the descent of other essential functions may and does follow different tempos and modes of evolution (Doolittle and Brunet, 2016). Analysis of the Syn3.0 construct highlighted genes of the Firmicutes/Tenericutes clade that descend from a variety of horizontal gene transfers. Being strict fermentors, the Tenericutes possess a highly derived form of ATP synthase likely to work as an ATPase to provide outside protons for vectorial transport of metabolites. In the case of translation, we have seen that the fate of ribosomal protein L27 differs in the Firmicutes/Tenericutes from that in other clades.

To go beyond this chassis, we now look for structural components specific to this clade and that identify functions carried over by different structures in other bacterial clades. DNA synthesis and replication are cases in point. The structure of DNA polymerase III splits bacteria into several consistent domains that must have evolved separately, exchanging genes back and forth by horizontal gene transfer. While subunit DnaE generally co‐evolved with the core of the translation machinery, the PolC subunit has a completely different tree of evolution, co‐evolving with other proteins that are generally absent from organisms which replicate in the absence of this subunit (Engelen *et al*., 2012). Even within the gamma‐Proteobacteria, often proposed as a consistent clade, one observes considerable differences between organisms: while *E. coli* has only a single DnaE type (DnaE1), *Pseudomonas putida* displays four different DNA polymerase III subunits, three descendants of DnaE (DnaE1, DnaE2 and DnaE3) and a PolC‐derived subunit (Timinskas *et al*., 2014). Interestingly, the second DnaE variant comes in, as a heterologous subunit of the enzyme, when the length of the genome sequence increases. Furthermore, the presence of DnaE2 together with DnaE1 is linked to bacteria featuring large GC‐rich genomes and living in aerobic environments.

To go beyond these observations, we explored the Syn‐3.0 unknowns that co‐evolved with subunit PolC of DNA polymerase III (Table 1), a feature that is common to the organisms of the A+T‐rich Firmicutes/Tenericutes clade. This allowed us to identify some of the conserved functions that must be found in other clades, while looking for different structural descents. Interestingly, more than half of the persistent proteins that were not identified by Engelen *et al*. (2012) were still unknown in the Syn3.0 annotation (Table 1). For example, small protein YyzM (MMSYN1_0873) displays some features of nucleic acid binding. It is worth exploring whether it could be a member of the replication machinery. By contrast, except for nanoRNase, that we discussed previously, well‐identified components form the RNA degradosome (ribonuclease M5, ribonuclease Y, ribonuclease J1). They highlight a structural specificity of A+T‐rich Firmicutes as compared with Enterobacteria (Danchin, 2009a).

**Table 1:** Proteins of Mycoplasma mycoides JCVI Syn3.0 co‐evolving with DNA polymerase PolC

Protein	Label	Function	References	Syn3.0 Category
PolC	MMSYN1_0303	DNA polymerase, alpha subunit, Firmicutes‐type	22333191	Equivalog
RnmV/YabF	MMSYN1_0003	Ribonuclease M5, ribosomal RNA 5S maturase	11976317	Equivalog
Rny/YmdA	MMSYN1_0359	Ribonuclease Y	23504012	Equivalog
RlmFO	MMSYN1_0434	m5U1939 modification in 23S rRNA (FAD‐tetrahydrofolate‐dependent)	24939895	Equivalog
HolA/YqeN	MMSYN1_0826	DNA polymerase III, delta subunit	23525462	Probable
HolB/YaaS	MMSYN1_0044	DNA polymerase III, delta prime subunit	23525462	Probable
RnjA/YkqC	MMSYN1_0600	Ribonuclease J1	23504012	Probable
EcfA/YbxA	MMSYN1_0643	Energizing coupling factor ABC multiple influx transporter (ATP‐binding protein)	26052893	Probable
EcfAB/YbaE	MMSYN1_0642	Energizing coupling factor ABC multiple influx transporter (ATP‐binding protein)	26052893	Probable
EcfT/YbaF	MMSYN1_0641	Permease component of the EcfAB influx transporters	26052893	Probable
NupO/RnsA/	MMSYN1_0010	Guanosine transporter, ATP‐binding protein, possibly promiscuous	21926227	Putative
FakB/YviA/YitS	MMSYN1_0616	Lipid salvage, fatty acid kinase subunit	25002480	Putative
NupP/RnsC	MMSYN1_0009	Guanosine transporter, permease, possibly promiscuous	21926227	Putative
NrnA/YtqI	MMSYN1_0139	nanoRNase	19553197	Generic
TrmK/YqfN	MMSYN1_0408	tRNA (adenine(22)‐N(1))‐methyltransferase	27098549	Generic
YloV	MMSYN1_0420	Enzyme of unknown specificity, possibly binding AdoMet and RNA	24939895	Generic
YloU	MMSYN1_0421	Similarity to yeast S‐adenosylmethioninedependent tRNA (uracil‐5‐)‐methyltransferase	10864043	Unknown
PdeB/YmdB	MMSYN1_0431	2′3′ and 3′5′ cyclic nucleotide monophosphates phosphodiesterase	21856853	Generic
YhaM	MMSYN1_0437	3′‐5′ oligonucleotide nuclease	19553197	Generic
DnaD	MMSYN1_0352	DNA remodelling primosomal protein, N‐terminus dimerization domain	23909787	Generic
YtpR	MMSYN1_0615	tRNA‐binding protein	17881821	Generic
RlmBB/YsgA	MMSYN1_0838	23S rRNA 2′‐O‐ribose G methyltransferase, modification of tRNA as well?	12377117	Generic
WhiA	MMSYN1_0817	Regulator of transcription/initiation of cell division	24097947	Generic
YtjP	MMSYN1_0493	Dipeptidase/deacylase	7528082	Generic
TrmNF/YabB	MMSYN1_0043	tRNA1(Val) (adenine(37)‐N6)‐methyltransferase	24809820	Generic
TrmK/YqfN	MMSYN1_0408	tRNA (adenine(22)‐N(1))‐methyltransferase	27098549	Generic
YlxM	MMSYN1_0430	Component of the signal recognition particle (SRP) protein membrane‐targeting pathway	24659773	Generic
YneF	MMSYN1_0317	Membrane spanning; conserved PP dipeptide, protein palmitoyltransferase?	19416851	Unknown
RppA/YsxB	MMSYN1_0500	L27N‐terminal protease, cleaves off the first nine residues of L27	25388641	Unknown
RulR/YlxR	MMSYN1_0421	RNA molecular ruler cofactor	26771646	Unknown
EttM/YfmM	MMSYN1_0853	Energy‐sensing regulator of translation	24389466	Unknown
YyzM	MMSYN1_0873	Nucleic acid binding protein, putative replication factor	21348639	Unknown

In the former clade, the universal methylation of tRNA U54 differs from that in most other organisms. TrmFO, the methylating activity, depends on methylenetetrahydrofolate rather than S‐adenosylmethionine (AdoMet). Interestingly, however, in *Mycoplasma capricolum,* in contrast to *B. subtilis*, the structural counterpart of TrmFO does not modify tRNA but specifically modifies m5U1939 in 23S rRNA, a conserved methylation catalysed by AdoMet‐dependent enzymes in all other characterized bacteria (Lartigue *et al*., 2014). The Mcap0476 methyltransferase (renamed RlmFO) represents the first folate‐dependent flavoprotein seen to modify ribosomal RNA using N5,N10‐methylenetetrahydrofolate as the one‐carbon donor. Here, we propose that this is also the case in Syn3.0 with the protein identified as TrmFO (MMSYN1_0434) being in fact RlmFO (Table S1). This will, however, ask for another activity for modification of U54 of tRNAs. Among proteins possibly involved in nucleic acid wielding, YloU (MMSYN1_0421, perhaps in conjunction with YloV, MMSYN1_0420) has weak similarity with yeast S‐AdoMet‐dependent tRNA (uracil‐5‐)‐methyltransferase and it might play this role. The whole families of TrmFO and RlmFO protein functions should be explored in depth, in particular in relation with co‐evolution with YloU‐YloV. We must note, however, that if this activity is indeed demonstrated it will require a redox activity allowing preservation of tetrahydrofolate, including within Syn3.0.

Another interesting nanomachine co‐evolving with PolC is a family of transporters, namely the energizing coupling factor (ECF) transporters. ECF transporters are composed of four subunits: a membrane‐embedded substrate‐binding subunit (EcfS), a transmembrane coupling subunit (EcfT) and two ATP‐binding‐cassette ATPases (EcfA and EcfA’). The three subunits associate to a variety of specificity subunits that allow transport of specific substrates, mostly vitamins. Several of the membrane proteins with unknown functions are likely to code for such specificity subunits. Among those, MMSYN1_0822 may be the FolT component of the folate transporter, as most important residues identified in its 3D structure are conserved (Xu *et al*., 2013). Beside ECF transporters, another family of transporters also co‐evolved with PolC: NupO and NupN are the ATP‐binding subunit and permease of a high‐affinity guanosine transporter (Belitsky and Sonenshein, 2011). Finally, we could speculate that membrane bound MMSYN1_0317, similar to *B. subtilis* YneF, with a conserved diproline, could be involved in acylating with long chain fatty acids relevant membrane proteins.

Now that we have a consistent picture of the Mycoplasma paleome, how could we go forward? We expect different kludges, such as the ribosomal protein L27 maturation process, to appear in different clades. Mycoplasmas as well as other members of the Tenericutes make a small fraction of bacteria. They are eroded A+T‐rich Firmicutes (Wolf *et al*., 2004). Among many of their ancestral features, the genome streamlining process has allowed them to dispense from iron metabolism. This is a highly specific trait that is likely fairly modern, deriving from a long process of evolution and perhaps inappropriate for the construction of versatile chassis. To be sure, iron is almost universally present in living organisms, and competition for iron is a major player in the formation of bacterial communities. Without iron, the cells cannot respire, making more difficult the formation of a proton gradient across the membrane. It is therefore likely that other synthetic chassis will use ATP synthase in its anabolic form rather than as an ATPase using ATP generated by fermentation, to build up a proton gradient. Many important metabolic differences will be expected in relation with this different set‐up. In particular, the extent of the flow within the core glycolytic pathway, with its related side reactions will considerably differ (see variations in *P. putida*, e.g. Nikel *et al*., 2015). The consequence is that variations on the theme of three‐carbon phosphorylated metabolites (with four, five or six carbons in particular) will be considerable. The related metabolites will impact the activity of the central carbon flow, and this will need to be regulated, including via dephosphorylation (Collard *et al*., 2016), as we discussed previously. The matching set‐up will differ in different chassis, and we should be extremely cautious in the prediction of the actual function of phosphatases, until authentic biochemical data are available. Other major differences should also be observed in parallel with different metabolic setups. For example, there is considerable difference within the gamma‐Proteobacteria (*E. coli*, cf *Buchnera* sp., *P. putida*,* Acinetobacter baylyi*, etc.) and with other clades such as that of the ancestor of mitochondria (alpha‐Proteobacteria).

Further specifications must also be taken into account. Bacteria are divided at least into monoderms, with an envelope comprising a single membrane, and diderms with two membranes and a periplasm (Zuckert, 2014). This will impact not only the process of protein secretion and export but also the division machinery. We can expect a variety of essential functions associated to the relevant paleome, differing from those found in Syn3.0. In general, except for a protein similar to the Zur regulator, we did not identify regulators. Are they necessary? Permeases are usually efficient, and this needs to be regulated by specific efflux (Danchin, 2009b). Furthermore, especially for chassis without a strong envelope, osmotic shock is likely to be extremely deleterious, asking for emergency valves such as mechanosensitive channels (Kocer, 2015) or at least aquaporins (Finn and Cerda, 2015). Also, while we hinted at some functions associated to these processes, we should consider more in depth what happens during stationary phase. A major riddle is that of the generation of a young progeny. How does the cell cope with its aged components, proteins in particular? This entails functions that, like Maxwell's demons, can separate between young and aged components, generating a young cell (Binder and Danchin, 2011). The NTP‐dependent chaperones and proteases identified in Syn3.0 are likely to play this role, but is this enough? Long‐term evolution of this particular chassis will probably tell, but we still need further models to fully understand the basic functions that make life.

## Conflicts of interest

The authors declare that they have no conflicts of interest associated to this work.

## Supporting information


**Table S1.** Prediction of unknown functions in *Mycoplasma mycoides* Syn3.0. The relevant protein names are indicated with reference to the standard annotation of Bacillus subtilis (). The Mycoplasma name is as in Hutchison et al. 2016. Predicted function is as indicated, associated with a suitable handle for exploration of the data. References at PubMed are provided as a PubMed identifier. Finally, the category is as proposed in Hutchison et al, 2016.
